# Effectiveness of a home-environmental intervention package and an early child development intervention on child health and development in high-altitude rural communities in the Peruvian Andes: a cluster-randomised controlled trial

**DOI:** 10.1186/s40249-022-00985-x

**Published:** 2022-06-06

**Authors:** Néstor Nuño, Daniel Mäusezahl, Jan Hattendorf, Hector Verastegui, Mariela Ortiz, Stella M. Hartinger

**Affiliations:** 1 Department of Public Health and Epidemiology, Swiss Tropical and Public Health Institute, Kreuzstrasse 2, CH-4123 Allschwil, Switzerland; 2 University of Basel, Petersplatz 1, CH-4001 Basel, Switzerland; 3 Unidad de Investigación en Desarrollo Integral, Ambiente y Salud, Universidad Peruana Cayetano Heredia, Av. Honorio Delgado 430, Urb. Ingeniería, S.M.P., Lima, Peru; 4 Programa Nacional Cuna Más, Lima, Peru

**Keywords:** Child development, Clinical trial, Diarrhoea, Improved biomass cookstoves, Peru, WASH

## Abstract

**Background:**

Unsafe drinking water, poor sanitation and hygiene, exposure to household air pollution and low cognitive and socio-emotional stimulation are risk factors affecting children in low- and middle-income countries. We implemented an integrated home-environmental intervention package (IHIP), comprising a kitchen sink, hygiene education and a certified improved biomass cookstove, and an early child development (ECD) programme to improve children´s health and developmental outcomes in the rural high-altitude Andes of Peru.

**Methods:**

We conducted a one-year cluster-randomised controlled trial among 317 children < 36 months divided into 4 arms (IHIP + ECD, IHIP, ECD, and Control) and 40 clusters (10 clusters per arm). ECD status (socio-emotional, fine and gross motor, communication, cognitive skills, and an overall performance) measured with the Peruvian Infant Development Scale and the occurrence of self-reported child diarrhoea from caretakers were primary outcomes. Secondary outcomes included the occurrence of acute respiratory infections and the presence of thermo-tolerant faecal bacteria in drinking water. The trial was powered to compare each intervention against its control arm but it did not allow pairwise comparisons among the four arms. Primary analysis followed the intention-to-treat principle. For the statistical analysis, we employed generalised estimating equation models with robust standard errors and an independent correlation structure.

**Results:**

We obtained ECD information from 101 children who received the ECD intervention (individually and combined with IHIP) and 102 controls. Children who received the ECD intervention performed better in all the domains compared to controls. We found differences in the overall performance (64 vs. 39%, odd ratio (*OR*): 2.8; 95% confidence interval (*CI*): 1.6–4.9) and the cognitive domain (62 vs 46%, *OR*: 1.9; 95% *CI*: 1.1–3.5). Data analysis of child morbidity included 154 children who received the IHIP intervention (individually and combined with ECD) and 156 controls. We recorded 110,666 child-days of information on diarrhoea morbidity and observed 1.3 mean episodes per child-year in the children who received the IHIP intervention and 1.1 episodes in the controls. This corresponded to an incidence risk ratio of 1.2 (95% *CI*: 0.8–1.7).

**Conclusions:**

Child stimulation improved developmental status in children, but there was no health benefit associated with the home-environmental intervention. Limited year-round access to running water at home and the possible contamination of drinking water after boiling were two potential factors linked to the lack of effect of the home-environmental intervention. Potential interactions between ECD and home-environmental interventions need to be further investigated.

*Trial registration:* ISRCTN, ISRCTN-26548981. Registered 15 January 2018—Retrospectively registered, https://doi.org/10.1186/ISRCTN26548981.

**Graphical abstract:**

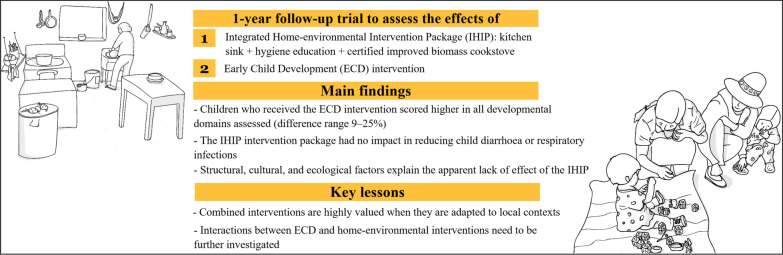

**Supplementary Information:**

The online version contains supplementary material available at 10.1186/s40249-022-00985-x.

## Background

A significant proportion of children living in low- and middle-income countries (LMIC) are highly exposed to health and environmental risk factors that often stem from unhealthy environments. In 2012, environmental risks in LMIC contributed to about 26% of deaths in children < 5 years [1]. In 2016, diarrhoea and respiratory infections combined accounted for more than one million under-five deaths globally [2, 3]. In addition, it is estimated that one third of children in the world within this age group have poor cognitive and/or socio-emotional development [4].

In Peru, diarrhoea and acute respiratory infections (ARI) are important causes of under-five morbidity in poor rural areas. In 2016, the incidence of diarrhoea in children < 5 years in the country was 2.13 cases per person [2] and the incidence of ARI 124.4 cases per 1,000 people [3]. Factors linked to increased levels of child diarrhoea and ARI were living in households with unsafe water, sanitation and hygiene (WASH) facilities and traditional biomass cookstoves [5]. Likewise, large cognitive development disparities persist between Peruvian children from different socio-economic quintiles [6].

For decades, individual low-cost interventions were implemented to reduce the burden of disease caused by diarrhoea and to improve children’s cognitive and psychosocial development [7, 8]. Integrating early child development (ECD) and home-environmental interventions is promising as they both target young children within the household environment [9, 10]. However, evidence on the benefits of combining several home-based interventions is still limited since sometimes the effects of interventions implemented simultaneously remain smaller than when they are applied individually [11, 12].

We present the results of a cluster-randomised controlled trial (henceforth referred to as “IHIP-2”) evaluating an integrated home-environmental intervention package (IHIP) and an early child development (ECD) programme that aimed to improve health and developmental outcomes in children under 36 months living in rural Andean Peru.

## Methods

### Setting

The trial was conducted in 82 rural Andean communities (registered populated centres) from the San Marcos and Cajabamba provinces, Cajamarca region, northern Peru. Both sites were high-altitude resource-limited locations. The majority of the population were small-scale farmers living in households with adobe walls, and using traditional biomass stoves or open fires for cooking. A detailed description of the study setting is found in Hartinger et al. [13].

### Study design

We implemented a cluster-randomised controlled trial to evaluate two home-based interventions: (i) a home-environmental package comprising a kitchen sink, hygiene education, and a certified improved biomass cookstove (ICS) (henceforth referred to as “IHIP”); and (ii) an ECD programme (henceforth referred to as “ECD”). The design led to four potential experimental conditions: (i) IHIP & ECD (henceforth referred to as “IHIP + ECD”), (ii) IHIP, (iii) ECD, and (iv) Control. A detailed description of the study design is found in Hartinger et al. [13].

The interventions comprising the IHIP were selected based on the results of a previous trial in the region [14, 15]. We selected the ICS model after a comprehensive community consultation [16]. Stoves were built with local materials and sinks were purchased locally. Participants received monthly visits during follow-up to reinforce hygiene education and the correct maintenance of ICS. The hygiene education component conveyed three main messages: (i) keeping kitchen environments clean; (ii) washing of mother and child’s hands with soap at key moments (e.g., before preparing meals); and (iii) household water treatment. We promoted boiling, since it was the method endorsed by local health authorities.

For the ECD intervention, we adapted the home-visiting component of the Peruvian National ECD programme (“Programa Nacional Cuna Más”—PNCM). Women living in the participating communities (mother facilitators—MFs) were trained to conduct weekly play-oriented, semi-structured activities with study children in the presence of their caretakers [17].

The study included families that (i) had at least one child < 1.5 years living at the household; (ii) used solid fuels as their main energy source for cooking/heating; (iii) had access to piped water in the yard; iv) did not plan to move within the next 24 months; and (iv) did not participate (but met inclusion criteria to do so) in the PNCM.

### Sample size calculation

We assumed three episodes of diarrhoea per child-year in the control arm, and a 25% reduction in incidence in the treatment arm. With 10 person-years of follow-up in each cluster and a coefficient of variation of 0.2, we estimated that 16 clusters for the intervention and control arm were sufficient to detect the anticipated reduction of incidence with a power of 80% at the 5% two-sided significance level. For the ECD intervention, we used the ECD outcome (percentage of tasks solved above the mean of the study population) of our previous intervention study [18] and assumed 60% above mean for the intervention and 40% above mean in the control arm. Using the equivalent formula for proportions, we calculated that 15 clusters for intervention and control were sufficient to detect the differences in ECD status with a power of 80% at the 5% two-sided significance level. To account for potential loss to follow-up, we included a total of 40 clusters (10 clusters per arm) in the study. The trial was sufficiently powered to compare each intervention against its control arm but it did not allow pairwise comparisons among the four arms. A detailed description of the sample size calculation, which followed the formula proposed by Hayes and Bennett [19], is found in Hartinger et al. [13].

### Randomisation and masking

Details on randomisation and masking are provided in Hartinger et al. [13]. In brief, the enrolled communities were aggregated into community-clusters based on their proximity to each other. We used a covariate-based constrained randomisation when allocating the communities into the four study arms [20]. First, clusters were divided into 8 strata of 4 clusters and 1 stratum of 8 clusters. Then, we generated two million random allocation sequences and selected those for which the maximum difference between arms fulfilled certain criteria (e.g., number of children, median community size or access to electricity within the community). Of the 164 allocation sequences that fulfilled all criteria, one was randomly selected. Given the trial design and nature of the interventions, the study was not blinded. Contamination of control communities was mitigated given intervention and control communities were geographically separated. Also, we conducted monthly household visits to identify possible structural changes in control households (i.e., installation of sinks). The ECD intervention required home visits and the use of age-adapted toys; contamination of control homes were considered unlikely to occur.

### Recruitment

**Fig. 1:**
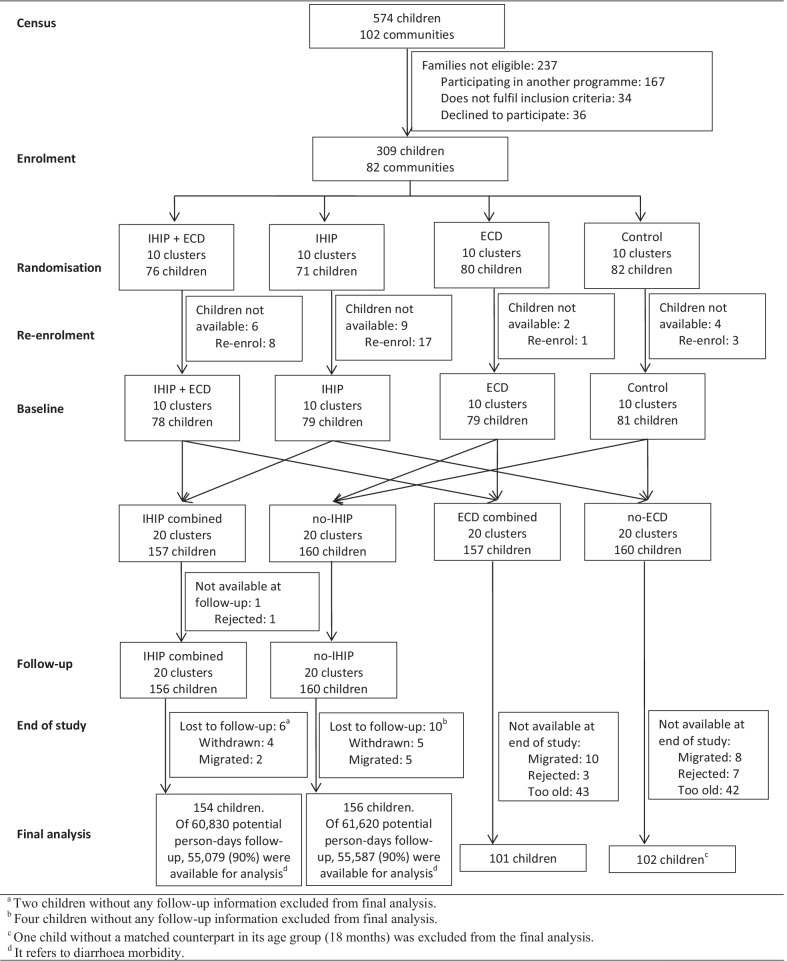
Flowchart of the cluster-randomised controlled trial. aTwo children without any follow-up information excluded from the final analysis. ^b^Four children without any follow-up information excluded from the final analysis. ^c^One child without a matched counterpart in its age group (18 months) was excluded from the final analysis. ^d^It refers to diarrhoea morbidity

We carried out a census in 2015 in collaboration with local health authorities to identify potential communities, children and pregnant women in their second and third trimester. Participants were enrolled between September 2015 and January 2016. From the screening census, we identified 102 communities with 574 potential children. During enrolment, 237 families were no longer eligible. We re-enrolled participants between January and February 2016 because 21 families were not available or declined to participate in the project at the beginning of the follow-up. A group of seven trained fieldworkers supervised by the field coordinator team enrolled participants. In total, 317 households from 82 communities participated in the trial (Fig. 1). A detailed description of the enrolment procedure is found in Hartinger et al. [13].

### Procedures

The IHIP-2 trial was conducted between April 2016 and May 2017. We visited all households weekly and collected daily and weekly self-reported information from the mother or caretaker about the occurrence of signs and symptoms of diarrhoea and ARI. During household visits, we measured respiratory rate, heart rate, and oxygen saturation in blood (SpO_2_) with portable pulse oximeters. Severely ill children were referred to local healthcare facilities for further evaluation. We collected health and anthropometric data monthly from participant’s clinical records at local health centres. When assessing ECD status at end-of-study, we applied two instruments i) the nationally validated Peruvian Infant Development Scale (ESDI) [21] and the Spanish version of the internationally validated Bayley Scales of Infant and Toddler Development (BSID) [22]. The ESDI tool was designed by the PNCM based on developmental studies conducted by the World Health Organization (WHO) and the recommendations of an expert panel. It assesses ECD status of children aged 1 to 36 months through direct observation, interaction or caregiver’s self-reports [21]. At end-of-study, some children were over the maximum age of 36 months at which age the ESDI tool can be carried out. The main reason was that the start of the trial needed to be postponed due to the re-enrolment. The BSID tool evaluates ECD status of children aged 1 to 42 months using a series of play-tasks [22]. The application of the ESDI allows the results to be used by the PNCM in its effort to expand the programme nationwide. We applied the BSID tool for comparability reasons despite differences between tools in age range, as the BSID is the current gold standard for measuring ECD status. A group of four fieldworkers conducted the ESDI assessments. They received a one week training from PNCM experts and were supervised by the field coordinator team. The BSID tool was applied by another group of four psychologist from the Universidad Peruana Cayetano Heredia (UPCH). Their work was supervised by a head psychologist who had previous experience applying the BSID. Information was revised on a daily basis to reduce the chance of missing data. We carried out weekly spot-check observations and obtained maternal self-reports to assess compliance with the IHIP, and monthly maternal self-reports for the ECD intervention. We collected 24 h stationary air pollution data in the kitchen using carbon monoxide (CO) monitors (EL-USB-CO, LASCAR Electronics, Whiteparish, England) and fine particulate matter (PM_2.5_) devices (APROVECHO-5000, APROVECHO Research Center, Cottage Grove, USA) at a one-meter distance from the ICS and at standard breathing height (1.5 m). Household air pollution (HAP) data were obtained from a sub-sample of 40 participants (henceforth referred to as “sentinel sub-sample”) on five occasions (before ICS installation, three times during follow-up, and at end-of-study). Water samples were obtained from the child’s main drinking source. They were collected at baseline and end-of-study for all study participants and in the sentinel sub-sample on three additional occasions during follow-up. We used a membrane filtration method for identifying thermo-tolerant faecal bacteria (DelAgua Water Testing Ltd, Marlborough, UK). All yellow colony-forming units were considered positive for bacteria growth, and microbial contamination was determined applying the WHO standards of zero viable coliforms. Finally, we administered a socio-economic questionnaire at baseline and end-of-study to assess household demographics, education and economic characteristics. We used the nationally validated Young Lives Wealth Index to classify participant’s wealth status [23]. A detailed description of the study’s methodology, field operations and procedures is found in Hartinger et al. [13].

### Outcomes

Diarrhoea and ECD status were the primary outcomes. We defined diarrhoea following the WHO definition of the passing of at least three loose stools within 24 h. We considered an episode to begin on the first day of diarrhoea and to end on the last day of passing a diarrhoeal stool, followed by at least three consecutive diarrhoea-free days. We defined ECD outcomes as the age standardised mean scores of socio-emotional, fine and gross motor, communication, and cognitive skills and an overall performance, defined as the arithmetic mean of the five categories. Secondary outcomes included: (i) ARI, defined (according to WHO standards) as presence of cough and fever. We defined an ARI episode to begin on the first day with cough and fever, ending on the last day with symptoms followed by at least seven symptom-free days; (ii) severe cases of diarrhoea, defined as persistent diarrhoea (14 days) or bloody diarrhoea; (iii) kitchen levels of CO and PM_2.5_ in the sentinel sub-sample; (iv) presence of thermo-tolerant faecal bacteria in drinking water samples; (v) stunting and underweight, defined according to WHO standards; and (vi) compliance linked to the interventions. We defined stove compliance as keeping the ICS structure and chimney in good condition (i.e., without deep cracks and not dissembled) and observing ICS use at the time of the visit or reporting ICS use in the last 24 h. Sink compliance was defined as keeping the structure in good condition and observing the presence of soap or dishes on the sink at the time of the visit. We defined ECD compliance as a reported ECD session since the last supervision visit.

We specifically selected diarrhoea and ECD status as primary outcome due to the important effort made in the international community to explore the effects of hygiene and ECD interventions combined [8, 12]. On the other hand, we selected ARI as a secondary outcome due to the low incidence of respiratory diseases detected in the area in previous studies [10], and the available evidence on the effect of ICS to detect improvements in children's health [24].

Safety of the interventions was assured; the ICS was certified by the Peruvian National Training Service for the Construction Industry (certificate number: 04-2015-LCM-GIN-SENCICO), and the ECD intervention followed the PNCM protocol. Parents were instructed to consult the nearest health centre for any health concern. Health centre contacts were assessed at each round of household visits and recorded if they were related to the trial intervention.

### Statistical analysis

Data were entered in the CSPro 6.1 database (U.S. Census Bureau, ICF International, Serpro S.A.) and cleaned, prepared and analysed the data using STATA 15.0 (Stata Corporation, College Station, TX, USA) and R 3.4 (R Foundation for Statistical Computing). For diarrhoea and ARI outcomes, we compared number of episodes and illness days. Because the scores of the ECD outcomes might be age dependent, we first calculated the mean score separately for each age category and dichotomised the outcome as performance above or below the age specific mean. For water samples, we compared the total number of samples with positive thermo-tolerant faecal bacteria.

To account for potential correlation within clusters, we employed generalised estimating equation (GEE) models with robust standard errors and an independent correlation structure. The correlation structure was pre-specified and not data driven. It has been suggested that independent correlation structures provide more robust results (compared to the exchangeable) if the number of participants varies among clusters, as was the case in our study [25]. For binary outcomes, we used the binomial family with logit link. For count data (number of episodes), we used a negative binomial distribution with log link and the natural logarithm of the number of days under observation as offset variable. The unadjusted model included the design factors and intervention effect. Further models were adjusted for child’s age and sex. All models were pre-specified in the trial protocol. Other baseline characteristics were of demographic nature. They were included in the covariate-based constrained randomisation but not in the adjusted statistical model. The primary analysis was performed according to the intention-to-treat principle using the full analysis set, i.e., all randomised and re-enrolled children with at least one day of follow-up information. We used the available case population for the analysis. No imputation of missing data was performed due to the low proportion of missing data for diarrhoea related outcomes (only 2.5% of children with no follow-up data) and because the proportion of missing data was balanced among trial arms.

## Results

### Participant characteristics

Initially, 82 communities with 309 children were randomised to the four trial arms (ratio 1:1:1:1). Before baseline assessment, 21 children were lost and 29 new eligible children were identified. In total, 317 children participated in the trial. At end-of-study, 85 children were too old to carry out the ESDI. One child was excluded from the analysis as no matched counterpart could be found in its age group. We obtained ESDI information from 101 children who received the ECD intervention (individually and combined with IHIP) and from 102 children acting as controls (henceforth referred to as “ECD combined” and “no-ECD” arms).

From the 317 children who participated in the study, 157 received the IHIP (individually and combined with ECD) and 160 acted as controls (henceforth referred to as “IHIP combined” and “no-IHIP” arms). Seven children without any follow-up data were excluded from the final analysis. The morbidity data analysis included 154 children from the IHIP and 156 from the no-IHIP arm. Information on diarrhoea morbidity was collected for 110,666 child-days in both arms, representing 90% of the total possible time-observations (Fig. 1).

At baseline, the four arms were balanced in most of the demographic, health, household, and wealth characteristics. Mothers were of similar mean age and had similar levels of primary education and years of schooling. The two-week prevalence of diarrhoea, cough, and fever, and anthropometric status were similar across arms. The majority of households had adobe walls, earthen floors, and tiled roofs. Participants predominantly belonged to the lowest wealth quintiles (Table 1). In addition, trial arms were well balanced with respect to the wealth index (see Additional file 1).

**Table 1:** Baseline demographic, health, household, and wealth characteristics of study participants from San Marcos and Cajabamba, Andean Peru, 2016

		IHIP + ECD		IHIP		ECD		Control
	*n*	Mean (*SD*) or % (*n*)	n	Mean (*SD*) or % (*n*)	*n*	Mean (*SD*) or % (*n*)	n	Mean (*SD*) or % (*n*)
Demographic characteristics	78		79		79		81	
Child sex (female)		43.6 (34)		50.6 (40)		53.2 (42)		55.6 (45)
Child age (years)^a^		1.5 (0.5)		1.5 (0.5)		1.7 (0.5)		1.7 (0.5)
Child < 1 year^a^		20.5 (16)		20.3 (16)		10.1 (8)		9.9 (8)
Age of caretaker (years)		27.3 (7.1)		28.4 (7.4)		27.2 (6.6)		28.0 (6.9)
Maternal education	78		79		79		81	
Years of schooling^b^		6.1 (3.5)		6.1 (3.3)		6.9 (3.5)		5.6 (3.2)
Children’s health outcomes	78		79		79		81	
Diarrhoea, two-week prevalence		18.4 (14)		22.8 (18)		19.0 (15)		26.3 (21)
Fever, two-week prevalence		21.8 (17)		21.5 (17)		25.3 (20)		23.5 (19)
Cough, two-week prevalence		35.1 (27)		31.7 (25)		39.2 (31)		29.1 (23)
Anthropometrics^c^	78		79		79		81	
Height-for-age, Z-scores	64	− 1.3 (1.1)	50	− 1.0 (0.9)	64	− 1.5 (1.1)	61	− 1.4 (0.8)
Weight-for-age, Z-scores	64	− 0.4 (1.0)	51	− 0.1 (0.8)	65	− 0.6 (0.9)	61	− 0.5 (0.9)
Household characteristics	78		79		79		81	
Adobe wall type		89.7 (70)		92.4 (73)		93.7 (74)		100 (81)
Earthen floor type		83.3 (65)		89.9 (71)		94.9 (75)		93.8 (76)
Roof tile type		82.1 (64)		87.3 (69)		86.1 (68)		95.1 (77)
Wealth classification	78		79		79		81	
1. Quintile (lowest)		28.2 (22)		24.1 (19)		17.7 (14)		14.8 (12)
2. Quintile		21.8 (17)		17.7 (14)		20.3 (16)		18.5 (15)
3. Quintile		14.1 (11)		21.5 (17)		26.6 (21)		19.8 (16)
4. Quintile		15.4 (12)		13.9 (11)		19.0 (15)		27.2 (22)
5. Quintile (highest)		20.5 (16)		22.8 (18)		16.5 (13)		19.8 (16)

*ECD* Early child development, *IHIP* Integrated Home-environmental Intervention Package, *SD* Standard deviation

^a^Age calculated for the start of follow-up (April 1, 2016)

^b^Higher education included the following categories: higher (non-university) education not completed: 12.5 years; higher (non-university) education completed: 14 years; university education not completed: 13.5 years; and university education completed: 16 years

^c^As estimated on December 15, 2015 (± 31 days). If several estimates within the range were available, the one closest to the date was selected

### Early child development and diarrhoea morbidity

Children from the ECD combined arm obtained better scores in all ESDI domains (difference range: 9–25%) (Table 2). The highest differences were observed in the overall performance and the cognitive domain (Table 3). The BSID evaluation showed small improvements in the ECD combined arm (see Additional file 2).

**Table 2:** Descriptive statistics of primary outcomes (ECD and child diarrhoea morbidity) in San Marcos and Cajabamba, Andean Peru, 2016

ESDI domains	Class parameter	ECD combined (*n* = 101)	no-ECD (*n* = 102)
Socio emotional	% (*n*)	66.3 (67)	56.9 (58)
Fine motor skills	% (*n*)	53.5 (54)	44.1 (45)
Gross motor skills	% (*n*)	60.4 (61)	47.1 (48)
Communication	% (*n*)	63.4 (64)	50.0 (51)
Cognitive	% (*n*)	62.4 (63)	46.1 (47)
Overall performance	% (*n*)	64.4 (65)	39.2 (40)

BSID domains^a^		ECD combined (*n* = 135)	no-ECD (*n* = 134)
Overall performance	Mean (*SD*)	9.0 (1.6)	8.6 (1.4)

Diarrhoea illness		IHIP combined (*n* = 154)	no-IHIP (*n* = 156)
Total days under observation	Total	55,079	55,587
Days under observation	Median (*IQR*)	379 (363, 385)	377 (362, 384)
Total number of episodes	Total	200	169
Episodes	Median (*IQR*)	1.0 (0.0, 2.0)	1.0 (0.0, 2.0)
Length of episode (days)	Mean (*SD*)	2.8 (1.9)	2.9 (2.5)
Total days with diarrhoea	Total	544	474
Days with diarrhoea	Median (*IQR*)	2.0 (0.0, 5.0)	1.0 (0.0, 4.5)
Days with diarrhoea	Mean (*SD*)	3.5 (5.0)	3.0 (5.0)
Diarrhoea incidence (*n* Episodes/child-year)^b^	Mean	0.4	0.3
Diarrhoea prevalence (*n* Days spend ill/child-year)^b^	Mean	1.2	0.9

*ECD* Early child development, *ESDI* Peruvian Infant Development Scale, *BSID* Bayley Scales of Infant and Toddler Development, *IQR* Interquartile range, *SD* Standard deviation

^a^The number of children is higher as the BSID is performed up to 42 months of age

^b^Per 100 child-year

**Table 3:** Effect of trial interventions on ECD, child diarrhoea, ARI, and water contamination outcomes. San Marcos and Cajabamba. Andean Peru, 2016

Outcome (n = 310)	Crude model^a^			Adjusted model^b^		
	*IRR/OR*	95% *CI*	*P*-value	*IRR/OR*	95% *CI*	*P*-value
Primary outcome						
Number of diarrhoea episodes (*IRR*)	1.2	0.8–1.7	0.36	1.1	0.8–1.6	0.45
Socio emotional (*OR*)	1.5	0.9–2.4	0.09	–	–	–
Fine motor skills (*OR*)	1.5	0.8–2.8	0.25	–	–	–
Gross motor skills (*OR*)	1.7	0.9–3.1	0.08	–	–	–
Communication (*OR*)	1.7	0.9–3.2	0.09	–	–	–
Cognitive (*OR*)	1.9	1.1–3.5	0.03	–	–	–
Overall performance (*OR*)	2.8	1.6–4.9	< 0.01	–	–	–
Secondary outcomes						
Diarrhoeal episodes with blood (*IRR*)	1.0	0.4–2.7	0.99	1.0	0.4–2.7	0.98
Days with diarrhoea (*OR*)	1.2	0.8–1.7	0.47	1.1	0.7–1.7	0.59
Number of ARI episodes (*IRR*)	0.9	0.7–1.2	0.63	0.9	0.7–1.2	0.67
Days with ARI (*OR*)	0.9	0.7–1.2	0.62	0.9	0.7–1.2	0.63
Thermo-tolerant bacteria (*OR*)	0.7	0.4–1.3	0.28	0.7	0.4–1.3	0.29

*ARI* Acute respiratory infection, *ECD* Early child development; *CI* Confidence interval, *GEE* Generalised estimating equation, *IRR* Incidence risk ratio, *OR* Odd ratio

^a^Estimated by GEE models adjusted for within-cluster correlation. For ECD domains, the outcome itself is age adjusted

^b^Estimated by GEE models adjusted for child’s sex and age and within-cluster correlation

We calculated 200 diarrhoea episodes (1.3 mean episodes per child-year) in the IHIP combined arm and 169 episodes (1.1 mean episodes per child-year) in the no-IHIP arm. Episode lengths, incidence and prevalence were similar in both arms (Table 2). The statistical analysis did not yield differences between arms (Table 3).

### Secondary outcomes

The number of bloody diarrhoea episodes was similar between IHIP combined and no-IHIP arms, although the total days with bloody diarrhoea were higher in the no-IHIP arm. We calculated 200 ARI episodes (1.3 mean episodes per child-year) in the IHIP combined arm compared to 217 (1.4 mean episodes per child-year) in the no-IHIP arm. Episode lengths, incidence, prevalence, SpO_2_ assessments, and respiratory rate during ARI episodes were balanced between arms. From 286 water samples collected at end-of-study, 52% (*n* = 151) tested positive for thermo-tolerant bacteria, with a higher proportion of positive samples in the no-IHIP arm. Differences in average 24-h kitchen PM_2.5_ concentrations were pronounced at the first follow-up visit, but they disappeared at end-of-study. We did not observe any change in anthropometric status between arms at end-of-study (see Additional file 3). The statistical analysis of secondary outcomes did not yield differences between arms (Table 3).

### Compliance

The median percentage of compliance with the ECD intervention was 95.5 [interquartile range (*IQR*): 86.7, 96.7]. From an initial compliance of about 70%, levels gradually increased and remained stable over time. A steady decline appeared at end of the study, coinciding with the time at which some children reached the maximum age to continue in the PNCM. The median percentage of compliance with the ICS was 90.6 (*IQR*: 87.8, 93.0). Levels were stable during follow-up. The median rate of sink compliance was 70.5 (*IQR*: 62.7, 79.4). We observed a steady increase in sink use, approximately up until week 24. Thereafter levels of sink use remained stable at 70% until end-of-study (see Additional file 4). Water supply interruptions and rationing occurred occasionally throughout. Then, families used their sinks to store water. This practice was abandoned when water scarcity ceased (see Additional file 4). No compliance information was collected in weeks 39 and 48 and research activities were halted in weeks 17 and 30 due to public holidays.

## Discussion

We conducted a community-randomised controlled trial with 317 families in the high-altitude rural Peruvian Andes to evaluate the impact of two home-based interventions. The children who received the ECD intervention showed higher age standardised mean scores in all the domains assessed with the ESDI tool than did control children (difference range 9–25%). The highest differences were observed in the cognitive domain (*OR*: 1.9; 95% *CI*: 1.1–3.5) and in the overall performance (*OR:* 2.8; 95% *CI*: 1.6–4.9). The application of the BSID for comparison showed also minor improvements in the children who received the ECD intervention. The IHIP had no measurable impact reducing the number of diarrhoea and ARI episodes. Other environmental indicators, such as presence of thermo-tolerant bacteria in drinking water and kitchen contamination, corroborated IHIP findings. The proportion of missing data was extremely low for diarrhoeal related outcomes. For the child developmental outcomes, the proportion of missing data seemed to be extremely high but the main reason (in about 75% of the missing cases), was that the child was too old (> 36 months) to apply the ESDI tool at the end of follow-up. Despite not being eligible for the primary child development outcome, they were enrolled for assessing child developmental status with the secondary ECD tool (BSID). In our child development data, imputation might perform poorly, because the observed number of completed tasks had different meanings among different age categories and the outcome was calculated according to the specific age category. The proportion of missing data was well balanced among trial arms.

The impact of the ECD intervention was comparable to previous studies conducted in other LMIC and Peru. Other home-visiting ECD interventions in Bangladesh, Colombia, and Pakistan showed improvements in cognitive, receptive language, and motor skills [8, 26, 27]. In Peru, a national evaluation of the PNCM found differences in cognitive and communication domains and improvements in fine motor and socio-emotional skills [28]. In rural Andean Cajamarca, the national urban day-care ECD programme, adapted to household level, improved all ECD domains [18]. The primary difference to our trial was the approach used to implement the ECD intervention; we used local MFs requiring a minimal education at secondary grade level while the Cajamarca trial employed primary teachers as ECD educators. This difference in skilled educators could have reduced the overall impact in our intervention, but not the direction of its effects. Evidence suggest that the use of adequately trained women living in the communities (instead of external fieldworkers) enhances the sustainability and acceptance of large-scale ECD programmes [29] The effects of our ECD intervention assessed with the BSID tool were smaller, although outcome trends were similar. We believe this variation may be caused by the fact that the application of the BSID is more complex. In Colombia, the application of the BSID showed a high validity compared to other tools [30]. However, the urban population in this study strongly differs from our typical rural Andean population. Different studies also indicate that the BSID tool might underestimate rates of developmental delay in infants across cognitive, language, and motor domains [31].

The home-environmental intervention had no impact in reducing child diarrhoea. This result differs from a previous evaluation where the IHIP achieved a more prominent 22% (*OR*: 0.87; 95% *CI*: 0.6–1.1) reduction in the number of diarrhoea episodes [10]. In our trial, three potential factors may have resulted in the lack of difference in child diarrhoea between arms. First, the promotion of hand washing and water boiling practices was already widespread in the study setting as these home-hygiene behaviours formed part of the Peruvian health system's regular health communication messages. This may have increased hygiene behaviours in the non-IHIP arm. Second, continuous prolonged periods of water scarcity during follow-up forced all study arms to adopt similar hygiene behaviours. Water storage infrastructures (e.g., ponds, dams) in the setting were not sufficient to ensure continuous water supply in periods of drought. Third, testing for thermo-tolerant bacteria in drinking water samples indicated that the boiling method was not as effective as expected. At end-of-study, 52% of drinking water samples tested positive for thermo-tolerant bacteria and we did not find statistical differences between study arms. This finding corroborates with results from water samples collected during the last follow-up visit with the sentinel sub-sample. We found that 36% of boiled drinking water samples stored in closed storage containers (e.g., jars, teapots) were contaminated (see Additional file 5). Boiling is the most commonly method used in LMIC for treating drinking water at households [32]. However, unsafe storage and handling may lead to the reduction of its effectiveness due to re-contamination [33, 34].

In addition, we do not exclude that the ECD intervention may have had positive effects on health outcomes, improving overall health seeking behaviours. Some approaches emphasise the interconnection between ECD and health improvements [35], and different trials found associations between ECD and health. In Pakistan, large improvements in diarrhoea and ARI outcomes were reported during an ECD intervention [36]. In Kenya and Bangladesh, small improvements in children’s developmental outcomes were linked to the effect of WASH interventions [12, 37]. Recall bias for child diarrhoea could have led to an underreporting of the outcome. Our recall period was one week, but recent evidence suggest that this time might underestimate diarrhoea prevalence [38]. Finally, the existence of different local types of child diarrhoea may have affected the reporting of diarrhoea cases. A qualitative study conducted with the sentinel sub-sample described the existence of eight local types of child diarrhoea. Out of these, participants only associated one type with hygiene and the germ theory of disease [39]. In the questionnaires used in this trial, we adapted the WHO definition of diarrhoea.

The IHIP also failed to reduce the number of ARI episodes. These results can be linked to HAP measurements, which showed no differences between arms at end-of-study. Other trials found no positive effects of ICS in reducing respiratory infections in children [10, 40]. Only in Rwanda, reductions in the prevalence of reported child ARI were obtained, although the trial showed no effect on 48-h personal exposure to PM_2.5_ [41]. According to recent systematic reviews and meta-analysis, ICS have so far failed to demonstrate any effect on children’s respiratory diseases and HAP reductions according to WHO target levels [24, 42]. For this reason, interventions implementing clean fuels and cooking technologies, such as liquefied petroleum gas and electricity, should be considered in future interventions. Conversely, we found remarkably high and stable levels of ICS compliance compared to those of similar trials that reported a continuous decline in ICS use [40, 41]. We believe these levels are partly explained due to the participatory approach applied in selecting the ICS model; thus, we recommend future trials implementing cooking technologies to apply similar approaches.

Our study has some limitations. Because the limited sample size did not allow pairwise comparisons or analysis of intervention interactions, we were not able to evaluate whether the ECD intervention alone had positive effects on health outcomes. In addition, we believe that the use of several data collection tools might have resulted in a lack of sufficient attention to some components and some research fatigue among participants (i.e., apathy and indifference in their responses). It has been suggested that conducting diarrhoea surveillance visits every 4 weeks is more accurate and requires only small increase in sample size [43]. Future randomised controlled trials should consider these factors as potential sources of information bias [44].

Overall, the IHIP-2 trial demonstrates that national home-visiting ECD programmes can lead to important improvements in children’s developmental status without compromising health. This evidence is especially useful to the PNCM and policymakers as it demonstrates the potential effectiveness of large-scale home-visiting ECD programmes [45]. In addition, carrying out weekly assessments for a whole year allowed us to take into account seasonality dynamics and monitor potential contamination. This suggests that the lack of effects of the home-environmental package should not only be attributed to the trial design (e.g., recall bias for diarrhoea), as external structural, cultural, and ecological factors (e.g., limited access to running water or local types of child diarrhoea) may play an important role in the impact of interventions. Future randomised controlled trials should consider the application of systems science approaches to analyse the organisational, community, and structural processes that are required for broad and sustained uptake of complex evidence-based interventions. Without examining the factors and system dynamics that are critical to the effectiveness and the ability to implement and scale-up interventions, research efforts may make the mistake of dooming novel interventions with promising potential precipitously [46].

## Conclusions

We present the results of a cluster-randomised trial evaluating an integrated home-environmental intervention package and an early child development (ECD) intervention in children living in the high-altitude rural Peruvian Andes. The home-delivered ECD intervention improved child developmental status without compromising health. The lack of effect of the home-environmental package to reduce the number of diarrhoea infection episodes should not only be attributed to the study or intervention design, as external structural, cultural, and ecological factors played an important role in the correct and continuous use of the intervention. Our study shows that combined interventions are highly valued when they are adapted to local contexts. This may help policy-makers to scale-up interventions while ensuring acceptance. Potential synergies and interactions between ECD and home-based health and hygiene interventions need to be further investigated. Our results also point to the need for assessing the effects of alternative clean fuels and cooking technologies on child health.

## Supplementary Information

**Additional file 1: **Wealth index among trial arms.

**Additional file 2: **Developmental status of children using the Bayley Scales of Infant and Toddler Development tool.

**Additional file 3: **Descriptive statistics of secondary health outcomes of the trial.

**Additional file 4: ** Compliance with the trial interventions.

**Additional file 5: **Thermo-tolerant bacteria in drinking water samples from the sentinel component.

## Data Availability

The data and materials underlying this article will be shared on reasonable request to the corresponding author.

## Abbreviations

ARI: Acute respiratory infections
BSID: Bayley Scales of Infant and Toddler Development
*CI*: Confidence interval
CO: Carbon monoxide
ECD: Early child development
ESDI: Peruvian Infant Development Scale
GEE: Generalised estimating equation
HAP: Household air pollution
ICS: Improved Biomass Cookstove
IQR: Interquartile range
IHIP: Integrated Home-environmental Intervention Package
IRR: Incidence risk ratio
LMIC: Low- and middle-income countries
MFs: Mother facilitators
PM_2.5_: Fine particulate matter
*OR*: Odd ratio
PNCM: Peruvian National ECD intervention
*SD*: Standard deviation
SpO_2_: Oxygen saturation in blood
UPCH: Cayetano Herida University
WASH: Water, sanitation and hygiene
WHO: World Health Organization
