# Supplementary material for: Effectiveness of a home-environmental intervention package and an early child development intervention on child health and development in high-altitude rural communities in the Peruvian Andes: a cluster-randomised controlled trial
Source: Infect Dis Poverty. 2022 Jun 6;11:66. doi: 10.1186/s40249-022-00985-x (PMC9169326; doi:10.1186/s40249-022-00985-x)
Supplement: Supplementary file 1 — Additional file 1: Wealth index among trial arms. [file 40249_2022_985_MOESM1_ESM.docx]

**Wealth index among trial arms**

Figure S1 represents the average wealth index among trial arms. Groups were well balanced with respect to the wealth index

| **Figure S1.** Wealth index among trial arms |
| --- |
| 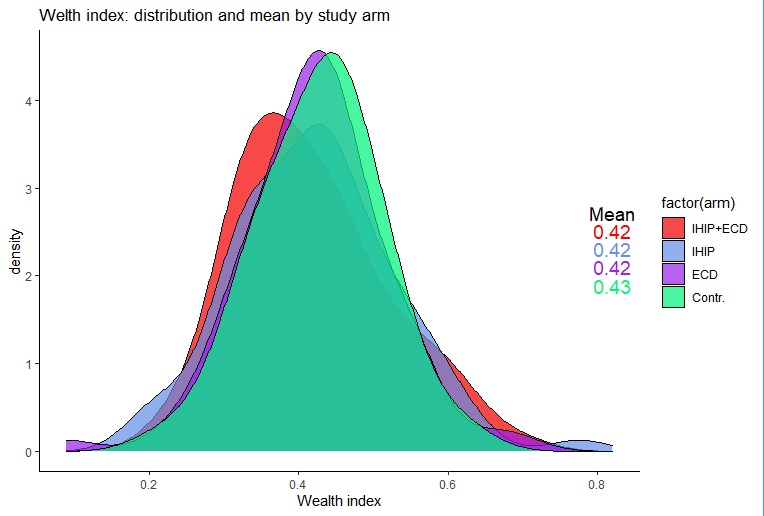 |
