# Supplementary material for: Effectiveness of a home-environmental intervention package and an early child development intervention on child health and development in high-altitude rural communities in the Peruvian Andes: a cluster-randomised controlled trial
Source: Infect Dis Poverty. 2022 Jun 6;11:66. doi: 10.1186/s40249-022-00985-x (PMC9169326; doi:10.1186/s40249-022-00985-x)
Supplement: Supplementary file 2 — Additional file 2: Developmental status of children using the Bayley Scales of Infant and Toddler Development tool. [file 40249_2022_985_MOESM2_ESM.docx]

**Evaluation of child developmental status using the Bayley Scales of Infant and Toddler Development**

We evaluated 271 children using the Bayley Scales of Infant and Toddler Development (BSID) tool. Scores ranged between 1–19 points. The median scalar scores in all the domains was balanced between groups: 9.0 (interquartile range (*IQR*): 8.0, 10.0) in the ECD arm and 9.0 (*IQR*: 7.0, 10.0) in the no-ECD arm. Children who received the ECD intervention obtained better mean scores in each domain, but results did not differ between arms (Table S2).

| **Table S2**. Developmental status of children using the BSID tool. San Marcos and Cajabamba. Andean Peru, 2016. | | | | | | |
| --- | --- | --- | --- | --- | --- | --- |
|  | ECD combined | | no-ECD | | Difference^a^ | Difference^b^ |
|  | *n* | Mean (*SD*) | *n* | Mean (*SD*) | estimate  *(95% CI)*^a^ | estimate  *(95% CI)*^b^ |
| *BSID domains*^c^ | 135 |  | 132 |  |  |  |
| Cognitive |  | 8.1 (1.5) |  | 7.8 (1.3) | 0.3 (-0.1–0.7) | 0.3 (-0.2–0.7) |
| Receptive communication |  | 9.0 (1.5) |  | 8.9 (1.2) | 0.2 (-0.2–0.6) | 0.2 (-0.2–0.6) |
| Expressive communication |  | 8.0 (1.9) |  | 7.8 (1.6) | 0.2 (-0.3–0.6) | 0.2 (-0.3–0.6) |
| Fine motor |  | 10.2 (2.4) |  | 9.7 (2.2) | 0.5 (-0.2–1.1) | 0.4 (-0.2–1.1) |
| Gross motor | 134 | 9.5 (3.3) |  | 9.0 (3.1) | 0.5 (-0.5–1.5) | 0.5 (-0.5–1.4) |
| ^a^ Model estimated by GEE models adjusted for within-cluster correlation.  ^b^ Model estimated by GEE models adjusted for within-cluster correlation, sex and age.  ^c^ The BSID tool as is performed up to 42 months of age.  BSID: Bayley Scales of Infant and Toddler Development; ECD: Early Child Development; GEE: Generalised Estimating Equations; *SD*: Standard Deviation; *CI*: Confidence interval. | | | | | | |
