# Supplementary material for: Effectiveness of a home-environmental intervention package and an early child development intervention on child health and development in high-altitude rural communities in the Peruvian Andes: a cluster-randomised controlled trial
Source: Infect Dis Poverty. 2022 Jun 6;11:66. doi: 10.1186/s40249-022-00985-x (PMC9169326; doi:10.1186/s40249-022-00985-x)
Supplement: Supplementary file 3 — Additional file 3: Descriptive statistics of secondary health outcomes of the trial. [file 40249_2022_985_MOESM3_ESM.docx]

**Descriptive statistics of secondary health outcomes**

Table S3 describes the secondary health outcomes of the trial.

| **Table S3**. Descriptive statistics of secondary outcomes (child diarrhoea morbidity, ARI, water contamination, household contamination and anthropometrics). San Marcos and Cajabamba. Andean Peru, 2016. | | | |
| --- | --- | --- | --- |
| *Diarrhoea illness* | Class Parameter | IHIP combined  (*n* = 154) | no-IHIP  (*n* = 156) |
| Persistent episodes | Total | 0 | 1 |
| Bloody episodes | Total | 9 | 9 |
| Days with bloody diarrhoea | Total | 16 | 27 |
| *ARI* |  | *n* = 154 | *n* = 156 |
| Total days under observation | Total | 55,153 | 55,681 |
| Days under observation | Median (*IQR*) | 381 (363, 386) | 378 (362, 384) |
| Total number of episodes | Total | 200 | 217 |
| Episodes | Median (*IQR*) | 1.0 (1.0, 2.0) | 1.0 (0.0, 2.0) |
| Length of episode (days) | Mean (*SD*) | 2.5 (2.0) | 2.5 (2.2) |
| Total days with ARI | Total | 455 | 492 |
| Days with ARI | Median (*IQR*) | 2.0 (1.0, 4.0) | 2.0 (0.0, 5.0) |
| Days with ARI | Mean (*SD*) | 3.0 (3.3) | 3.2 (3.6) |
| Days with ARI and difficulties breathing | Total | 82 | 109 |
| Total SpO_2_ measurements during ARI episode | Total | 22 | 22 |
| SpO_2_ during ARI episode | Median (*IQR*) | 92.0 (89.0, 95.0) | 94.0 (92.0, 96.0) |
| Total respiratory rate measurements during ARI episode | Total | 45 | 40 |
| Respiratory rate during ARI episode | Median (*IQR*) | 27.0 (24.0, 28.0) | 24.5 (22.5, 27.0) |
| ARI incidence (*n* Episodes/child-year)^a^ | Mean | 0.4 | 0.4 |
| ARI prevalence (*n* Days spend ill/child-year)^a^ | Mean | 0.9 | 0.9 |
| *Drinking water contamination* |  | *n* = 142 | *n* = 144 |
| Thermo-tolerant bacteria | % (*n*) | 48.6 (69) | 56.9 (82) |
| *24-hour kitchen PM_2.5_ contamination (µg/m^3^)* |  | *n* = 20 | *n* = 20 |
| First follow-up visit (June-November 2016) | Mean (*n*) | 82.9 (20) | 255.0 (13) |
| End of study visit (May-August 2017) | Mean (*n*) | 140.0 (19) | 121.0 (20) |
| *Anthropometrics^b^* |  | *n* = 146 | *n* = 148 |
| Height-for-age, Z-scores | Mean (*SD*) | -1.7 (1.0) | -1.7 (1.0) |
| Stunting | % (*n*) | 34.3 (50) | 36.5 (54) |
| Weight-for-age, Z-scores | Mean (*SD*) | -0.6 (0.9) | -0.7 (0.8) |
| Underweight | % (*n*) | 4.8 (7) | 6.1 (9) |
| ^a^ Per 100 child-year.  ^b^ Anthropometrics as estimated on 30^th^ of April 2017 ±31 days. If several estimates within the range were available, the one closest to the date was selected.  ARI: Acute respiratory infection; PM_2.5_: Fine particulate matter; IHIP: Integrated Home-environmental Intervention Package; *IQR*: Interquartile range; *SD*: Standard deviation; SpO_2_: Oxygen saturation in blood. | | | |
