# Supplementary material for: Effectiveness of a home-environmental intervention package and an early child development intervention on child health and development in high-altitude rural communities in the Peruvian Andes: a cluster-randomised controlled trial
Source: Infect Dis Poverty. 2022 Jun 6;11:66. doi: 10.1186/s40249-022-00985-x (PMC9169326; doi:10.1186/s40249-022-00985-x)
Supplement: Supplementary file 4 — Additional file 4: Compliance with the trial interventions. [file 40249_2022_985_MOESM4_ESM.docx]

**Compliance with the trial interventions**

Figure S4.1 represents the compliance with the improved biomass cookstove, kitchen sink and ECD intervention during the one-year follow-up

| **Figure S4.1.** Compliance with trial interventions |
| --- |
| 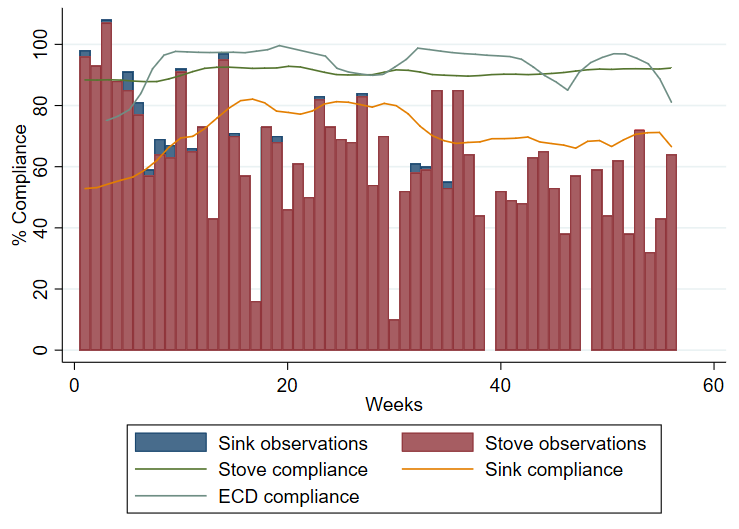 |

Figure S4.2 represents sink compliance from the arm using the home-hygiene intervention and rainfall levels (litres/m^2^) in three study districts (D1–D3) during trial´s follow-up. Information was extracted from the public records of the National Service of Meteorology and Hydrology of Peru. In the three districts analysed, levels of sink use followed the same pattern.

| **Figure S4.2.** Association between rainfall and sink use during follow-up |
| --- |
| 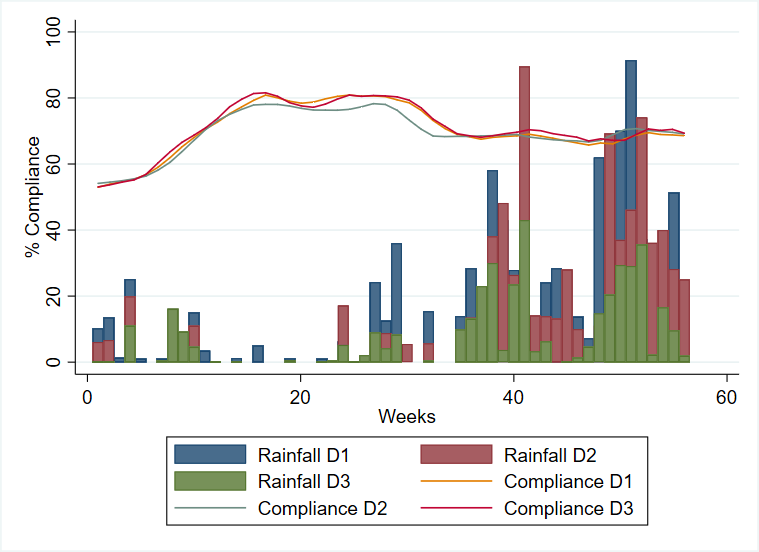 |
