# Supplementary material for: Effectiveness of a home-environmental intervention package and an early child development intervention on child health and development in high-altitude rural communities in the Peruvian Andes: a cluster-randomised controlled trial
Source: Infect Dis Poverty. 2022 Jun 6;11:66. doi: 10.1186/s40249-022-00985-x (PMC9169326; doi:10.1186/s40249-022-00985-x)
Supplement: Supplementary file 5 — Additional file 5: Thermo-tolerant bacteria in drinking water samples from the sentinel component. [file 40249_2022_985_MOESM5_ESM.docx]

**Water testing in the trial’s sentinel component**

We collected drinking water samples on five occasions during follow-up in a sub-sample of 40 participants (10 participants per arm). Water samples were obtained from the point-of-use, the container that families used to store boiled water, and the child´s main drinking source. Table S5 describes the results of the last follow-up visit (May-August 2017).

| **Table S5**. Thermo-tolerant bacteria in drinking water samples from the sentinel component. San Marcos and Cajabamba. Andean Peru, 2016. | | | | | | |
| --- | --- | --- | --- | --- | --- | --- |
|  | IHIP combined | | no-IHIP | | Total | |
|  | *n* | % (*n*) | *n* | % (*n*) | *n* | % (*n*) |
| Point-of-use | 19 | 42.1 (8) | 20 | 30.0 (6) | 39 | 35.9 (14) |
| Source (tap water) |  | 98.5 (17) |  | 95.0 (19) |  | 92.3 (36) |
| Boiled water^a^ | 10 | 50.0 (5) | 12 | 25.0 (3) | 22 | 36.4 (8) |
| Container (teapot) |  | 50.0 (5) |  | 58.3 (7) |  | 54.6 (12) |
| Child´s main drinking source | 19 | 42.1 (8) | 20 | 35.0 (7) | 39 | 38.5 (15) |
| ^a^ Only collected when caretakers reported having boiled drinking water stored at the time of the visit.  IHIP: Integrated Home-environmental Intervention Package. | | | | | | |
